# Investigation of the Mechanism of Complement System in Diabetic Nephropathy via Bioinformatics Analysis

**DOI:** 10.1155/2021/5546199

**Published:** 2021-05-24

**Authors:** Bojun Xu, Lei Wang, Huakui Zhan, Liangbin Zhao, Yuehan Wang, Meng Shen, Keyang Xu, Li Li, Xu Luo, Shasha Zhou, Anqi Tang, Gang Liu, Lu Song, Yan Li

**Affiliations:** ^1^Hospital of Chengdu University of Traditional Chinese Medicine, Chengdu, 610072 Sichuan, China; ^2^Key Laboratory of Chinese Internal Medicine of Ministry of Education and Dongzhimen Hospital, Beijing University of Chinese Medicine, Beijing 100700, China; ^3^Chengdu Seventh People's Hospital, Chengdu, 610213 Sichuan, China; ^4^Centre for Cancer and Inflammation Research, School of Chinese Medicine, Hong Kong Baptist University, Hong Kong; ^5^The First Affiliated Hospital of Guangxi University of Chinese Medicine, Nanning 530023, China

## Abstract

**Objectives:**

Diabetic nephropathy (DN) is a major cause of end-stage renal disease (ESRD) throughout the world, and the identification of novel biomarkers via bioinformatics analysis could provide research foundation for future experimental verification and large-group cohort in DN models and patients.

**Methods:**

GSE30528, GSE47183, and GSE104948 were downloaded from Gene Expression Omnibus (GEO) database to find differentially expressed genes (DEGs). The difference of gene expression between normal renal tissues and DN renal tissues was firstly screened by GEO2R. Then, the protein-protein interactions (PPIs) of DEGs were performed by STRING database, the result was integrated and visualized via applying Cytoscape software, and the hub genes in this PPI network were selected by MCODE and topological analysis. Gene Ontology (GO) and Kyoto Encyclopedia of Genes and Genomes (KEGG) pathway enrichment analyses were carried out to determine the molecular mechanisms of DEGs involved in the progression of DN. Finally, the Nephroseq v5 online platform was used to explore the correlation between hub genes and clinical features of DN.

**Results:**

There were 64 DEGs, and 32 hub genes were identified, enriched pathways of hub genes involved in several functions and expression pathways, such as complement binding, extracellular matrix structural constituent, complement cascade related pathways, and ECM proteoglycans. The correlation analysis and subgroup analysis of 7 complement cascade-related hub genes and the clinical characteristics of DN showed that C1QA, C1QB, C3, CFB, ITGB2, VSIG4, and CLU may participate in the development of DN.

**Conclusions:**

We confirmed that the complement cascade-related hub genes may be the novel biomarkers for DN early diagnosis and targeted treatment.

## 1. Introduction

Diabetic nephropathy (DN), the most common microvascular complication of diabetes, is becoming the leading cause of ESRD worldwide [1]. The typical clinical features of DN consist of the decreased glomerular filtration rate and persistent proteinuria [2]. Currently, there are several pathways reported to be involved in the pathogenesis of DN, including the activation of polyol and protein kinase C (PKC) pathway [3], the generation of advanced glycation end products (AGE) [4], and intraglomerular hypertension caused by glomerular hyperfiltration [5]. In addition, microinflammation and subsequent extracellular matrix (ECM) pathways are also involved in the progression of DN [6]. Furthermore, the complement system is reported to involve in the development of DN but mostly focused on the lectin pathway [7, 8]. There are few studies aimed to explore the potential significance of the classic and alternative complement pathways in the pathogenesis of DN at the transcriptional level [9]. However, the reported studies of these complement pathways are not comprehensive and detailed and other pathways of the complement system as well as their clinicopathological relevance with DN are still poorly understood. Taking together, the pathogenic and molecular mechanisms of DN have not been elucidated comprehensively yet, the prevalence rate of DN is high, treatment is difficult, and prognosis is poor [10, 11]. Therefore, new diagnostic biomarkers and novel therapies of DN should be further studied that will be beneficial for improving the clinical prognostic of DN.

Genome-wide transcriptome analysis using microarray and bioinformatics technology enable the identification of biomarkers for disease progression and gain insights into the disease pathogenesis and molecular classification [12, 13]. With the wide application of genome transcriptome analysis, a large amount of core slice data has been produced and stored in a public database, including the GEO database [14]. Recently, some microarray data analyses about DN have been carried out, and numerous differentially expressed genes (DEGs) have been identified. Researchers have used microarray data from DN models of different species to determine molecular mechanisms and genetic factors involved in DN [15, 16]. Previous bioinformatics analyses using human DN gene chip data (GSE47183) from the GEO database found that the VSIG4, CD163, C1QA, C1QB, MS4A6A, COL6A3, COL1A2, CD44, FN1, NPHS1, WT1, PLCE1, TNNT2, TNNI1, and TNNC1 genes played important roles in DN progression through ECM-receptor interaction, PI3K-Akt signaling pathway, focal adhesion, proteoglycans in cancer, and complement and coagulation cascades [17]. Yang et al. [18] identified hub genes associated with DN using GSE30528 chip data, including VEGFA, ITGA3, ITGB5, COL4A3, COL4A5, CBLB, and CCL19. In addition, the key miRNAs related to DN were predicted based on the hub genes, including miR-200b/c, miR-29a/b/c, miR-25, miR-27, miR-23, miR-181, miR-17, miR-506, and miR-124a. All these genes were mainly enriched in the ECM-receptor interaction and PI3K/Akt signaling pathways to initiate the pathogenesis of DN [18]. Liu et al. [19] carried out a weighted gene coexpression network analysis of GSE104948 chip data and discovered that FCER1G played a crucial role in the pathogenesis of DN.

**Figure 1:**
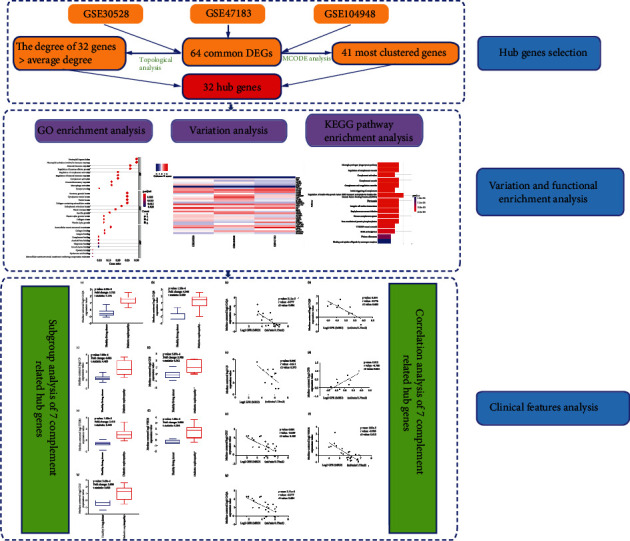
A flow chart based on an integration strategy of multiple-microarray analysis.

In this study, we reanalyzed three microarray datasets, GSE30528, GSE47183, and GSE104948; key biomarkers were identified by selecting the significant differentially expressed genes (DEG) between DN and normal glomerular samples. Then, biological processes and signaling pathways that participated in DN will be explored on basis of DEGs. The pathogenesis of DN was studied by GO and KEGG pathway enrichment as well as PPI network analysis. Additionally, the Nephroseq v5 online platform was used to analyze correlations and to perform subgroup analysis among the hub genes and clinical features of DN to further explore the pathogenesis, pathophysiological and molecular mechanisms involved in DN. In conclusion, a total of 64 DEGs and 32 hub genes were identified, which may be potential diagnostic biomarkers and therapeutic targets for preventing the occurrence and development of DN; the flow chart of this study is shown in Figure 1.

## 2. Methods and Materials

### 2.1. Microarray Data Analysis

We downloaded the datasets including GSE30528 [9], GSE47183 [20], and GSE104948 [16] from GEO (http://www.ncbi.nlm.nih.gov/geo) [21], an international public functional genomics data repository of next-generation sequence, chips, and microarrays. The DEGs in DN and normal renal tissues were firstly selected by the GEO2R online tool [22] with ∣LogFC | >1 and *P* < 0.05. Subsequently, the overlap DEGs among the three datasets were shown in the Venn diagram via using FunRich, a software widely used for gene and protein functional enrichment and interaction network analysis [23].

### 2.2. Protein-Protein Interaction (PPI) Network Construction, Topological Analysis, and Hub Gene Identification

A PPI network of overlapping DEGs was established based on the STRING platform (https://string-db.org/) [24] to retrieve nearly all functional interactions among the expressed proteins. Protein interaction information derived from the STRING database was imported into the Cytoscape software where the interaction information was integrated and visualized [25].

Then, the most significant modules in the PPI network were selected by molecular complex detection (MCODE) [26]; the parameters of clustering and scoring were MCODE score ≥ 2, degree cutoff = 2, node score cutoff = 0.2, max depth = 100, and *k*‐score = 2. At the same time, topological analysis was performed by using NetworkAnalyzer in Cytoscape software and four topological features (degree, betweenness centrality, average shortest path length, and closeness centrality) were analyzed [27]. Subsequently, selecting nodes with degrees higher than the average number, taking the intersection of the topological analysis, and clustering analysis result as the hub genes. If the LogFC < 0, the expression of the hub gene was deemed to be downregulated, while LogFC > 0, gene expression was deemed to be upregulated. Additionally, a network of the hub genes and their coexpression network was performed by using FunRich software.

### 2.3. GO and KEGG Pathway Enrichment Analysis

GO is a comprehensive and widely used database for the classification of gene functions, consisting of biological process (BP), molecular function (MF), and cell component (CC) [28]. KEGG (http://www.kegg.jp/) is an encyclopedia of genomes, which links genomic information with higher-order functional information to capture significantly enriched biological pathways [29]. In our study, GO functional annotation and KEGG pathway enrichment analysis were performed through applying the clusterProfiler package of R software, when *P* < 0.05 was deemed as a screening threshold.

### 2.4. The Association between Hub Genes and Clinical Features of DN

Correlation analysis and subgroup analysis between hub genes and clinical features were carried out via the Nephroseq v5 online tool (http://v5.nephroseq.org) [30] to confirm the potential functions of hub genes participated in DN.

### 2.5. Statistical Analysis

Pearson's correlation analysis between hub genes and glomerular filtration rate (GFR) in patients with DN was conducted via applying Nephroseq v5. Comparisons between two subgroups were carried out via an unpaired Student's *t*-test. All tests were two-tailed, with a *P* value < 0.05 considered statistically significant. The statistical analyses were performed by using GraphPad Prism (version 7.0; GraphPad Software, La Jolla, California).

## 3. Results

### 3.1. Identification of DEGs in DN

**Figure 2:**
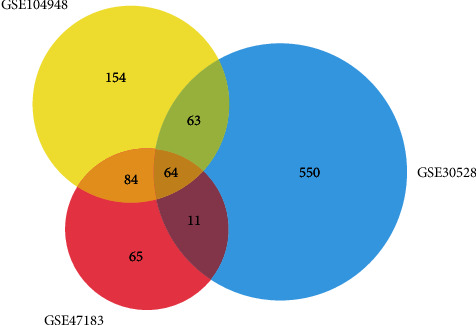
Overlapping DEGs of multiple microarrays.

We used the GEO database to search the gene expression profiles of GSE30528, GSE47183, and GSE104948 in DN and normal renal samples. These three datasets contain 51 normally functioning human glomerulus samples and 35 diabetic human glomerulus samples. These study samples were derived from healthy human transplant donors, diagnostic renal biopsies, and tumor-nephrectomy specimens. Then, the gene information obtained from the database was analyzed by GEO2R. The cutoff criteria were [LogFC] > 1 and *P* < 0.05. We found 224, 687, and 365 DEGs in GSE47183, GSE30528, and GSE104948 datasets, respectively. Subsequently, these DEGs from three datasets were imported into FunRich to identify the common DEGs; a total of 64 DEGs were detected (Figure 2).

### 3.2. PPI Network Analysis and Hub Gene Selection

**Figure 3:**
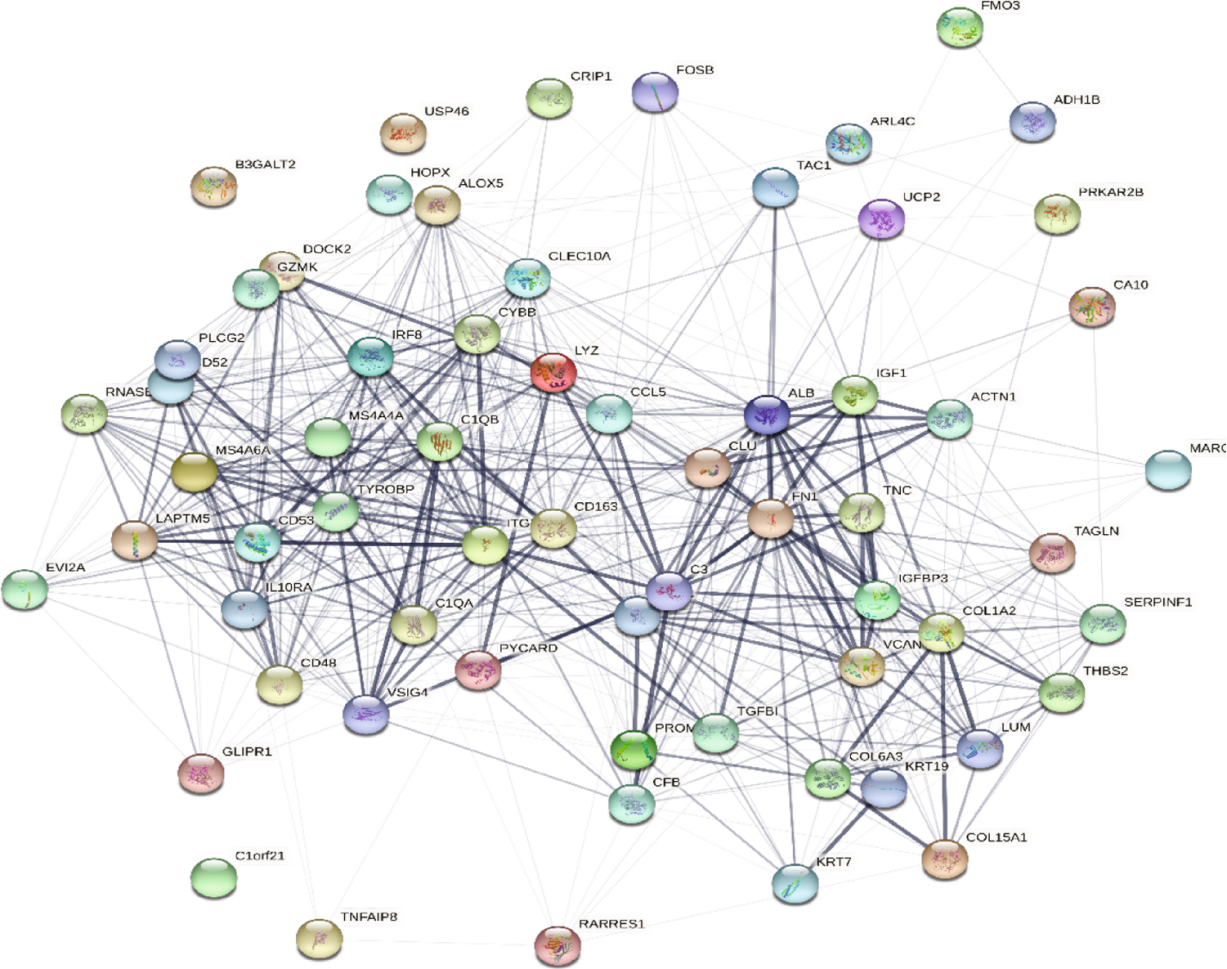
The PPI network of overlapping DEGs of three microarray datasets. Each circular node represents a protein target, and the 3D structure in the circular nodes shows the protein spatial structure. The lines among different nodes represent the association among potential protein targets, while the width of lines was according to the action intensity.

**Figure 4:**
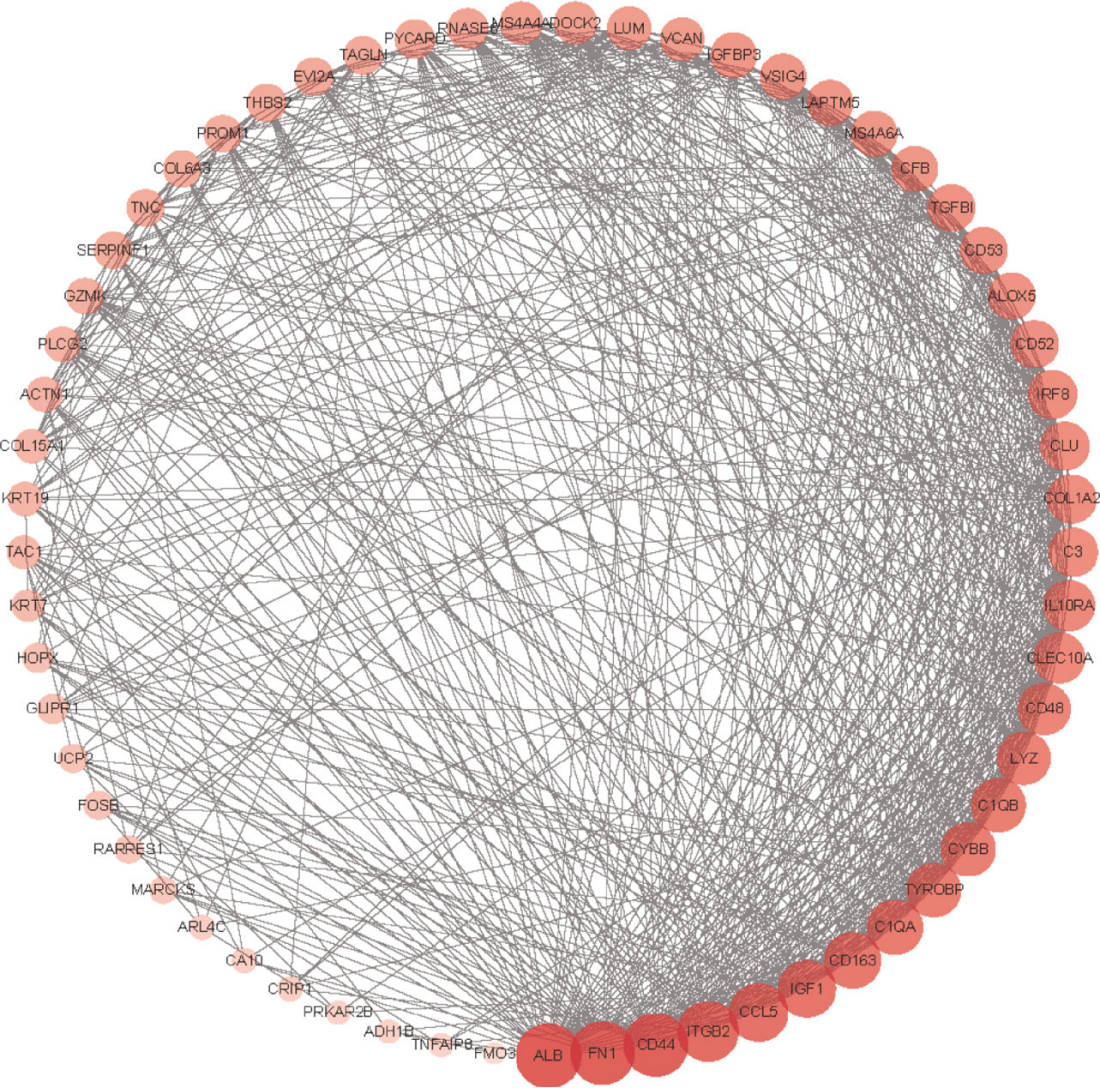
PPI network visualization and analysis.

A total of 64 common DEGs were imported into STRING online database to construct the PPI network, the interaction network was based on the selected targets with a medium confidence score of 0.15, and finally, 652 edges and 64 key nodes were embodied (Figure 3). PPI network of common DEGs was visualized by Cytoscape (Figure 4), and the three most significant modules were recognized by the MCODE plug-in of Cytoscape.

**Figure 5:**
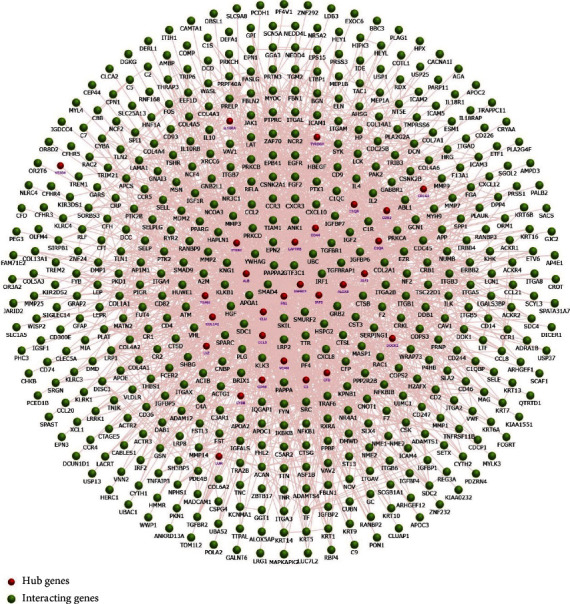
The coexpression network analysis of hub genes.

**Figure 6:**
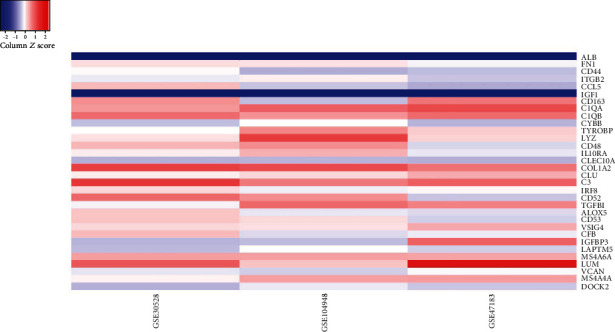
The heat map of the hub genes.

Among these three modules, a total of 41 DEGs were identified (Table 1). In addition, the average node degree is 21.377 after the topological analysis of common DEGs, we selected the genes higher than the average degree, taking the intersection of the genes higher than the average degree, and most significant clustering genes result as the hub genes. The hub genes included 2 downregulated genes (ALB and IGF1) and 30 upregulated genes (FN1, CD44, ITGB2, CCL5, CD163, C1QA, C1QB, CYBB, TYROBP, LYZ, CD48, IL10RA, CLEC10A, COL1A2, CLU, C3, IRF8, CD52, TGFBI, ALOX5, CD53, VSIG4, CFB, IGFBP3, LAPTM5, MS4A6A, LUM, VCAN, MS4A4A, and DOCK2), which are exhibited in Table 2. Furthermore, the coexpression network analysis of hub genes is displayed in Figure 5 and the heat map of the hub gene expression in the three GEO datasets is shown in Figure 6.

### 3.3. GO and KEGG Pathway Enrichment Analysis of Hub Genes in DN

**Figure 7:**
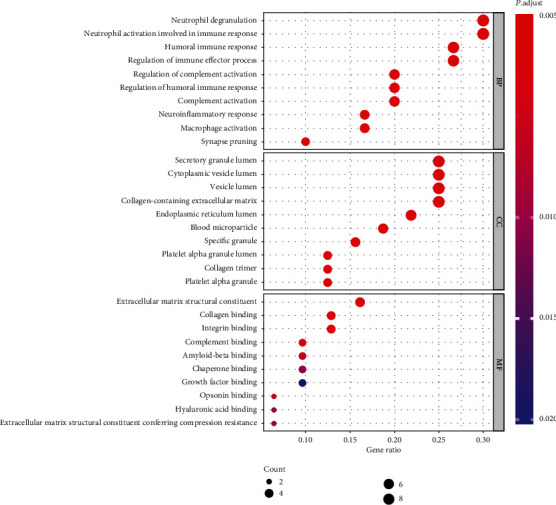
Enriched GO terms for BP, MF, and CC of hub genes. The color of the node is displayed in a gradient from red to blue according to the ascending order of the *P* value, while the size of the node is showed according to the ascending order of the number of gene counts.

After applying the clusterProfiler package for hub gene enrichment analysis, we selected the top 10 remarkably enriched BP terms for analysis, including regulation of complement activation, neutrophil degranulation, neutrophil activation involved in immune response, regulation of humoral immune response, humoral immune response, neuroinflammatory response, complement activation, regulation of immune effector process, macrophage activation, and synapse pruning. Besides, the top 10 CC terms were screened, consisting of secretory granule lumen, cytoplasmic vesicle lumen, vesicle lumen, blood microparticle, collagen-containing extracellular matrix, endoplasmic reticulum lumen, platelet alpha granule lumen, specific granule, collagen trimer, and platelet alpha granule. Furthermore, the top 10 MF terms were selected, containing collagen binding, complement binding, extracellular matrix structural constituent, integrin binding, opsonin binding, amyloid-beta binding, hyaluronic acid binding, extracellular matrix structural constituent conferring compression resistance, chaperone binding, and growth factor binding. These processes are of great significance to further understand the hub genes that participated in the progression of DN. The results of the GO analysis are illustrated in Figure 7.

**Figure 8:**
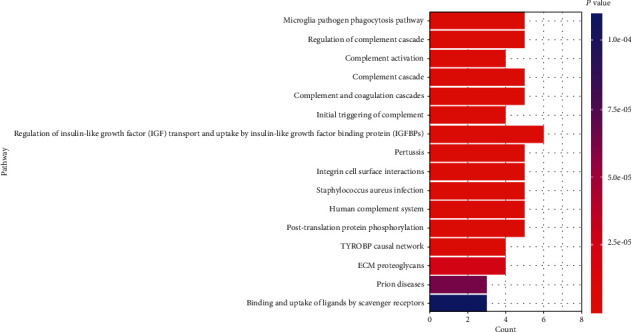
KEGG pathway analysis of hub genes. A total of 16 pathways are screened out according to the criteria of *P* < 0.05. The longitudinal axis displays the name of different pathways, and the transverse axis shows the count of enriched genes. In addition, the length of the bar is showed according to the ascending order of the number of gene counts, while the color of the bar is displayed in a gradient from blue to red according to the descending order of the *P* value.

After the KEGG pathway enrichment analysis, a total of 16 significantly enriched pathways were selected out based on the threshold of *P* < 0.05 (Figure 8). The results indicated that these genes were mainly associated with regulation of insulin-like growth factor (IGF) transport and uptake by insulin-like growth factor binding proteins (IGFBPs), posttranslational protein phosphorylation, ECM proteoglycans, complement and coagulation cascades, complement cascade, initial triggering of complement, regulation of complement cascade, human complement system, and complement activation. The results prove that hub genes derived from these three datasets may participate in the progression and development of DN by regulating complement cascade, insulin resistance, and inflammatory reaction. Among them, complement cascade-related pathways were most enriched.

### 3.4. Association between the Hub Genes and Clinical Features of DN

**Figure 9:**
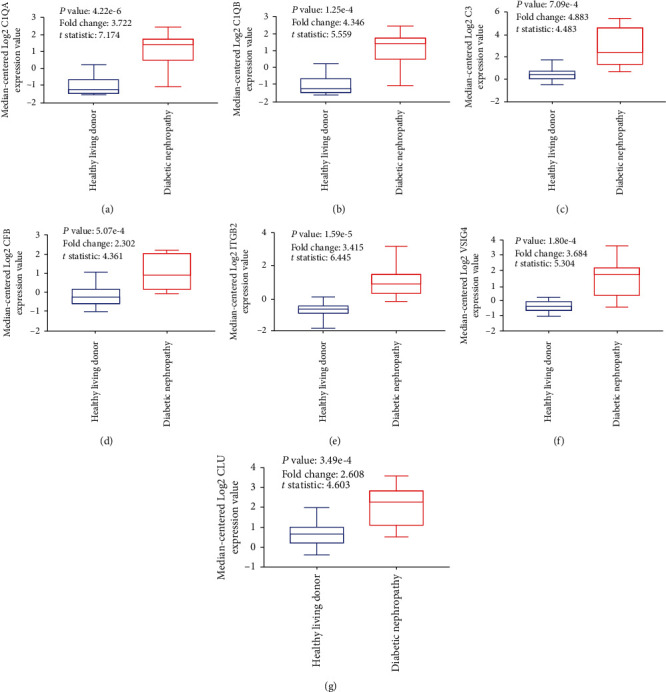
The different expressions of complement cascade-related hub genes (C1QA, C1QB, C3, CFB, ITGB2, VSIG4, and CLU) in DN renal tissues and healthy kidney tissues. (a) The expression of C1QA in DN renal tissues is higher than in healthy kidney tissues. (b) The expression of C1QB in DN renal tissues is higher than in healthy kidney tissues. (c) The expression of C3 in DN renal tissues is higher than in healthy kidney tissues. (d) The expression of CFB in DN renal tissues is higher than in healthy kidney tissues. (e) The expression of ITGB2 in DN renal tissues is higher than in healthy kidney tissues. (f) The expression of VSIG4 in DN renal tissues is higher than in healthy kidney tissues. (g) The expression of CLU in DN renal tissues is higher than in healthy kidney tissues.

**Figure 10:**
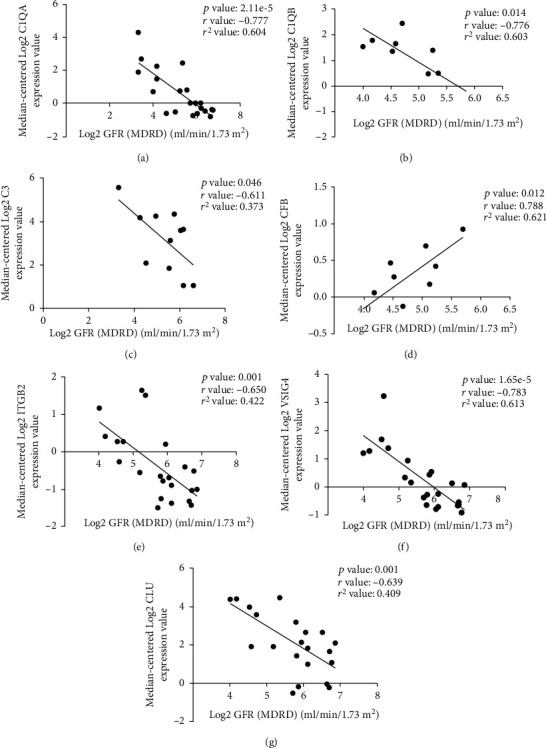
The correlation analysis between complement cascade-related hub genes (C1QA, C1QB, C3, CFB, ITGB2, VSIG4, and CLU) and the expression of GFR in DN patients. (a) The expression of C1QA was negatively correlated with GFR (*P* < 0.001, *r* = –0.777). (b) The expression of C1QB was negatively correlated with GFR (*P* = 0.014, *r* = –0.776). (c) The expression of C3 was negatively correlated with GFR (*P* = 0.046, *r* = –0.611). (d) The expression of CFB was positively correlated with GFR (*P* = 0.012, *r* = 0.788). (e) The expression of ITGB2 was negatively correlated with GFR (*P* = 0.001, *r* = –0.650). (f) The expression of VSIG4 was negatively correlated with GFR (*P* < 0.001, *r* = –0.783). (g) The expression of CLU was negatively correlated with GFR (*P* = 0.001, *r* = –0.639).

Of the overlapping DEGs identified in this study, 32 were recognized as hub genes. With the use of Nephroseq v5, the expression of complement cascade-related hub genes (C1QA, C1QB, C3, CFB, ITGB2, VSIG4, and CLU) showed the difference between DN patients and healthy living donors (Figure 9); we found all complement cascade-related hub genes were downregulated in the DN renal tissues compared with healthy kidney samples. In addition, the correlation between the complement cascade-related hub genes and GFR of DN patients was determined (Figure 10). The expression of CFB in DN renal tissue samples was positively correlated with GFR. Thus, the expression of CFB may contribute to the maintenance and improvement of renal function. The expression of the C1QA, C1QB, C3, ITGB2, VSIG4, and CLU was negatively correlated with GFR. Therefore, the expression changes of these six genes may result in the occurrence and development of DN.

## 4. Discussion

The prevalence of type 2 diabetes has risen dramatically worldwide, and DN is one of the most common complications of type 2 diabetes which has become the main cause of ESRD [31]. DN is featured as glomerular injury, glomerular hypertrophy, and glomerular basement membrane thickening [32]. Many cases of DN have a delayed diagnosis and are complicated to treat, which may contribute to the poor renal prognosis of patients with DN [33]. However, DN is the result of multiple gene interactions and the molecular mechanisms of DN remain poorly understood because of the complexity of the etiology differences [34]. Therefore, potential biomarkers for early diagnosis and targeted treatments are urgently needed. Currently, collagen binding and ECM-receptor interaction have already been verified to make a considerable contribution to the development of DN [35, 36]. In addition, there are two main complement cascade-associated mechanisms that have been reported to be involved in the development and progression of DN [37]. First, hyperglycaemia is considered to cause glycation of complement regulatory proteins which can lead to dysfunction of their regulatory capacity [38]. Second, the activation of the lectin pathway in response to glycated proteins is expressed on the surface of cells due to overexposure to glucose [39].

The development of microarray technology enables us to explore genetic alterations in DN and have a better understanding of the molecular mechanisms, eventually identifying the novel markers in DN [40–43]. In our study, a set of 64 overlapping DEGs from the GSE30528, GSE47183, and GSE104948 datasets were identified. Among them, the expression of 32 hub genes (30 upregulated genes and 2 downregulated genes) was selected for performing GO and KEGG enrichment analyses to explore the molecular mechanisms of hub genes involved in the development of DN. The GO enrichment analysis revealed that hub genes were involved in multiple biological processes, including regulation of complement activation, regulation of humoral immune response, humoral immune response, complement activation, regulation of immune effector process, and macrophage activation. The hub genes, such as C1QA, C1QB, C3, CFB, ITGB2, VSIG4, and CLU, can participate in complement cascade [44–47]. CD163, CD44, CD48, CD52, and CD53 may involve in humoral immune response and macrophage activation [48, 49]. Thus, the biological processes of hub genes are relatively consistent with the pathogenesis and mechanism of DN. Additionally, cellular components constitute secretory granule lumen, cytoplasmic vesicle lumen, vesicle lumen, blood microparticle, collagen-containing extracellular matrix, endoplasmic reticulum lumen, platelet alpha granule lumen, specific granule, collagen trimer, and platelet alpha granule. It indirectly elucidates the complexity of the pathogenesis of DN and its damage to several cellular components [5]. Moreover, molecular functions are mostly enriched in collagen binding, complement binding, extracellular matrix structural constituent, amyloid-beta binding, and extracellular matrix structural constituent conferring compression resistance. It reveals that hub genes may target these molecular functions to affect DN progression and it is consistent with previous studies [50].

After performing KEGG enrichment analysis, we found that hub genes were mainly enriched in the regulation of IGF transport and uptake by IGFBPs, ECM proteoglycans, and complement cascade-related pathways. ECM proteoglycans, one important ECM component, show a more complex pattern of changes in DN, which could be mediated by TGF-*β* [51]. IGF-IGFBP signaling components play an important role in the maintenance of normal renal function and the development of DN. IGF-I expression increases in the diabetic kidney in the autocrine/paracrine manner that promotes matrix production, mesangial cell proliferation, and migration, but this process can be opposed by IGFBPs [52].

Moreover, experimental and clinical evidence has showed that multiple components of the complement system involved in the pathogenesis of DN [37]. And we also found hub genes were mostly enriched in complement cascade-associated pathways, consisting of complement and coagulation cascades, complement cascade, initial triggering of complement, regulation of complement cascade, human complement system, and complement activation. In these 32 hub genes, we selected 7 complement cascade-related genes, including C1QA, C1QB, C3, CFB, ITGB2, VSIG4, and CLU. Among them, C3 is the central component in the complementary system which plays an important role in the classical complement pathway and alternative complement pathway [47, 53]. We found that C3 expression was higher in DN renal samples compared with healthy renal samples and was negatively correlated with GFR (*P* = 0.046, *r* = –0.611). In addition, the classic complement pathway was activated in DN renal tissues with significant increases of C1QA and C1QB mRNAs [47]. We discovered that C1QA and C1QB expressions were higher in DN renal samples and were negatively correlated with GFR ((*P* < 0.001, *r* = –0.777) and (*P* = 0.014, *r* = –0.776), respectively). CFB is a crucial factor for activating the alternative complement pathway [54], which is elevated in adipose tissue and serum from patients with type 2 diabetes [55], but the correlation between CFB and DN has not been illustrated yet. We found that CFB expression was higher in DN renal samples compared with healthy renal samples and was positively correlated with GFR (*P* = 0.012, *r* = 0.788). ITGB2 is implicated in the network linking complement system to T cell activation [56], but it also remains poorly understood about the relationship with DN. We found that ITGB2 expression was higher in DN renal samples and was negatively correlated with GFR (*P* = 0.001, *r* = –0.650). Besides, VSIG4 is a B7 family-related protein and acts as a complement receptor for C3 [57]; it has been reported that VSIG4 can induce the epithelial-mesenchymal transition of renal tubular cells when exposing to high-glucose condition [58], but how VSIG4 affect DN through the complement system is still unknown. We found that VSIG4 expression was higher in DN renal samples and was negatively correlated with GFR (*P* < 0.001, *r* = –0.783). CLU is a kind of complement regulatory protein which can bind to C5b-7 and inhibit the generation of membrane attack complex [59] and was found upregulated in the glomerular of both DN patients and streptozotocin-induced diabetic mice [46]. We found that CLU expression was higher in DN renal samples and was negatively correlated with GFR (*P* = 0.001, *r* = –0.639). Taking these findings together will offer novel potential targets in future DN research.

Independent microarray analysis always induces false-positive rates [60]. Due to the heterogeneity of the tissues or samples in independent studies or a single cohort study, gene array results are always either limited or inconsistent. In this study, we reanalyzed three microarray datasets (GSE30528, GSE47183, and GSE104948) and combined various bioinformatics methods and expression profiling techniques to identify candidate DEGs and predict hub genes in DN. In addition, we also used a clinical database (Nephroseq v5 platform) to analyze correlations and perform subgroup analysis among the hub genes and clinical manifestations of DN.

However, there are some limitations of this study. Firstly, the predictions above were not confirmed by experiments and are rather preliminary in silico studies; these are only useful for an initial screening. It is required to be validated by in vitro/vivo experiments and large-group cohort before any valid conclusion could be taken. Secondly, because of the heterogeneity of detailed demographic data, it is difficult to obtain the more convincing association between hub genes and the severity of diabetic glomerular injury using the samples from different datasets. Thirdly, this study had a relatively small sample size; in future studies, we also need to combine and analyze more clinical samples based on similar demographic data to validate these results.

## 5. Conclusions

The microarray and bioinformatics technology have provided new perspectives for researchers to study the potential molecular mechanisms and regulatory targets of DN. A total of 64 DEGs and 32 hub genes were identified as biomarkers for the clinical diagnosis of DN and as potential targets for novel treatments. However, predictions were not verified by experiments, and the number of samples used for analysis was limited. Thus, the specific molecular mechanisms and biological functions of hub genes need further exploration. In conclusion, our study found hub genes which might be involved in the pathogenesis of DN especially in complement cascade-related signaling pathways. At the same time, we also linked the expression of hub genes with the clinical manifestation of DN, which may provide the novel methodologies for DN early diagnosis and targeted therapies.

## Figures and Tables

**Table 1:** The most significant clusters of common DEGs.

Cluster	Network	Nodes	Edges	Score	Cluster genes
1	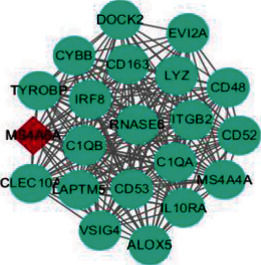	21	203	20.3	EVI2A, RNASE6, LAPTM5, LYZ, CD53, CLEC10A, TYROBP, DOCK2, ALOX5, IL10RA, C1QA, MS4A6A, CD52, C1QB, ITGB2, MS4A4A, CD48, CD163, VSIG4, CYBB, IRF8
2	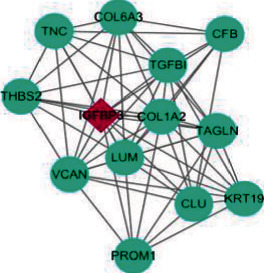	13	62	10.3	TGFBI, VCAN, THBS2, CFB, CLU, COL1A2, IGFBP3, COL6A3, LUM, KRT19, TNC, PROM1, TAGLN
3	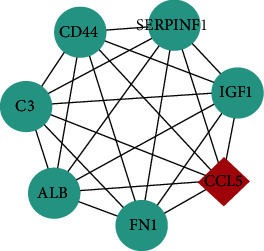	7	21	7	CD44, ALB, FN1, CCL5, SERPINF1, IGF1, C3

The red nodes display the core genes of the cluster; the green nodes represent the normal cluster genes. Furthermore, the density of lines among different nodes shows the interaction relationship between cluster genes.

**Table 2:** Hub genes of diabetic nephropathy.

Groups	Gene symbol	GSE30528		GSE104948		GSE47183	
		LogFC	*P* value	LogFC	*P* value	LogFC	*P* value
Up	FN1	1.540307	1.86*E* − 06	1.688679	6.53*E* − 14	1.411328	0.000115
	CD44	1.410913	0.00288	1.154713	1.03*E* − 09	1.118944	0.004117
	ITGB2	1.288601	0.000137	1.624558	1.71*E* − 09	1.176759	0.015233
	CCL5	1.720493	0.000615	1.281747	1.58*E* − 08	1.010635	0.002817
	CD163	1.973348	2.66*E* − 06	1.260515	4.52*E* − 05	2.25682	0.006126
	C1QA	1.922276	3.04*E* − 08	2.469202	7.39*E* − 08	2.643819	0.002315
	C1QB	2.204686	4.22*E* − 07	2.123409	8.65*E* − 08	2.331991	0.003483
	CYBB	1.082376	2.88*E* − 05	1.559454	6.84*E* − 10	1.081369	0.01398
	TYROBP	1.3831	0.000242	2.191158	1.95*E* − 08	1.720981	0.000578
	LYZ	1.529532	0.00258	2.79008	2.08*E* − 08	1.693295	0.000448
	CD48	1.736706	0.000071	2.1464	7.11*E* − 11	1.271107	0.002955
	IL10RA	1.468163	0.000276	1.928371	3.65*E* − 09	1.340032	0.004027
	CLEC10A	1.005234	6.1*E* − 06	1.197336	6.34*E* − 07	1.050224	0.032825
	COL1A2	2.590987	9.18*E* − 06	2.656871	2.14*E* − 07	2.275034	0.005026
	CLU	1.472253	0.000508	1.734574	1.59*E* − 06	1.912975	0.002851
	C3	2.679961	0.000157	2.281075	3.84*E* − 06	2.430408	0.017567
	IRF8	1.583247	0.000131	1.488973	1.09*E* − 06	1.493004	0.005593
	CD52	2.232654	9.78*E* − 05	2.140349	6.98*E* − 13	1.169746	0.007569
	TGFBI	1.32786	0.000642	2.396142	3.41*E* − 13	2.168562	3.43*E* − 05
	ALOX5	1.673735	0.00568	1.450238	7.51*E* − 09	1.277775	0.001606
	CD53	1.672055	5.65*E* − 05	1.74099	5.92*E* − 08	1.230233	0.012145
	VSIG4	1.576657	5.22*E* − 06	1.696336	1.31*E* − 07	1.919467	0.003
	CFB	1.720388	6.19*E* − 05	1.382782	5.74*E* − 05	1.365459	0.021665
	IGFBP3	1.033501	9.66*E* − 05	1.23278	0.000966	2.409502	0.002813
	LAPTM5	1.062031	0.00158	1.554621	9.52*E* − 08	1.238422	0.001673
	MS4A6A	1.869037	6.81*E* − 06	2.054775	9.74*E* − 09	1.959976	0.00497
	LUM	2.374619	4.49*E* − 05	1.843351	0.00363	3.496266	0.004413
	VCAN	1.273776	0.00177	1.33971	4.88*E* − 05	1.440879	0.034958
	MS4A4A	1.446755	2.65*E* − 05	2.039072	1.15*E* − 08	1.994681	0.00792
	DOCK2	1.009537	0.0028	1.457016	1.34*E* − 09	1.181051	0.003642

Down	ALB	-1.78623	0.00896	-2.38803	0.000092	-2.63068	0.007517
	IGF1	-2.57456	7.11*E* − 05	-1.6702	1.25*E* − 05	-1.63423	3.29*E* − 05

## Data Availability

The data used to support the findings of this study are included within the article.
